# Antifeedant Diterpenoids against *Tribolium castaneum* from the Stems and Twigs of *Ceriops tagal *(Rhizophoraceae)

**DOI:** 10.3390/molecules16076060

**Published:** 2011-07-20

**Authors:** Shu Shan Du, Cheng Fang Wang, Jing Li, Hai Ming Zhang, Qi Zhi Liu, Zhi Long Liu, Zhi Wei Deng

**Affiliations:** 1 Protection and Utilization of Traditional Chinese Medicine of Beijing Area Major Laboratory, Beijing Normal University, Haidian District, Beijing 100875, China; 2 Analytical and Testing Center, Beijing Normal University, Beijing 100875, China; 3 Department of Entomology, China Agricultural University, Haidian District, Beijing 100193, China

**Keywords:** feeding deterrents, *Ceriops tagal*, *Tribolium castaneum*, tagalsin

## Abstract

The screening of several Chinese mangrove plants for insecticidal principles showed that ethanol extract of *Ceriops tagal* stems and twigs possessed significant feeding deterrent activity against the red flour beetle, *Tribolium castaneum* (Family: Rhizophoraceae). From the ethanol extract, three feeding deterrent diterpenoids were isolated by bioassay-guided fractionation. The compounds were identified as tagalsin A, B, and H on the basis of their phytochemical and spectral data. Tagalsin A, B, and H exhibited strong feeding deterrent activity against *T. castaneum* adults with EC_50_ values of 375.3 ppm, 277.3 ppm, and 285.45 ppm, respectively.

## 1. Introduction

The red flour beetle [*Tribolium castaneum* (Herbst)] is one of the most widespread and destructive primary insect pests of stored cereals [1]. Infestations not only cause significant losses due to the consumption of grains; they also result in elevated temperature and moisture conditions that lead to an accelerated growth of molds, including toxigenic species [2]. Botanical pesticides have the advantage of providing novel modes of action against insects that can reduce the risk of cross-resistance as well as offering new leads for design of target-specific molecules [3,4]. Control of stored product insects relies heavily on the use of synthetic insecticides and fumigants, which has led to problems such as disturbances of the environment, increasing costs of application, pest resurgence, pest resistance to pesticides and lethal effects on non-target organisms in addition to direct toxicity to users [5]. These problems have highlighted the need for the development of new types of selective stored product pest-control alternatives. During a screening program for new agrochemicals from Chinese medicinal herbs and wild plants, ethanol extract of Chinese mangrove plant, *Ceriops tagal* (Perr.) C.B. Robinson stems and twigs (Family: Rhizophoraceae) were found to possess significant feeding deterrent activity against *T. castaneum*. This plant is well distributed in Southern China, Eastern Africa, and Oceania [6]. It is used as a folkloric medicine in China. The bark of *C. tagal* is a powerful astringent and is used in the treatment of hemorrhage in defecation. The oil of the breed is a kind of antipruritic and used in the treatment of acariasis and chillblain. The leaves, when boiled in water, are used as a substitute for quinine to heal paludism [7]. The bark of this plant has been used for the treatment of infected wounds in Thailand and for obstetric and hemorrhagic conditions in the Philippines [8]. The decoction of its leaves has been used for the treatment of malaria in China [9], whereas that of its bark has been utilized for the treatment of hemorrhage and malignant ulcers in India [10]. The chemical constituents and bioactivities of *C. tagal *have been extensively studied and the known chemical constituents of this medicinal herb include monoterpenoids, diterpenoids, triterpenoids, flavonoids, alkaloids, polyphenolics, cardiac glycosides, saponins and sterols [7,8,11,12,13,14,15,16,17,18,19,20,21]. However, the bioactive compounds against insects have not been isolated and identified from this plant. In this paper, we report the isolation and identification of three feeding deterrents contained in *C. tagal* stems and twigs against *T. castaneum* by bioassay-guided fractionation.

## 2. Results and Discussion

### 2.1. Isolated Bioactive Compounds

**Figure 1:**
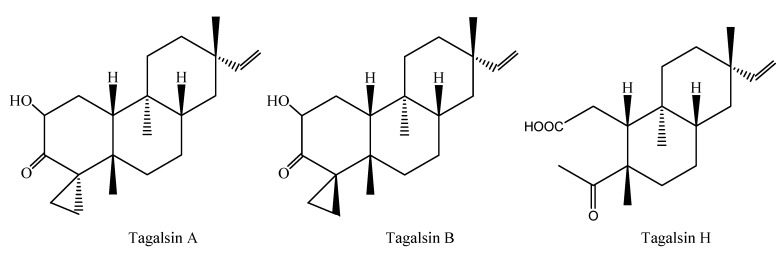
Structures of feeding deterrents isolated from *Ceriops tagal* stems and twigs.

Three bioactive compounds were isolated and based on bioassay-guided fractionation and identified based on their spectroscopic data and comparison with literature vales. Their chemical structures are given in Figure 1.

### 2.2. Feeding Deterrent Activity

The feeding deterrent activity of the three isolated compounds against the red flour beetle is shown in Table 1. The three pure compounds, tagalsin A, B and H exhibited significant feeding deterrent activity against *T. castaneum* adults at a concentration of 30 ppm and above in a concentration-dependent manner (Table 1). The concentration used in this study (30 ppm) to observe feeding deterrent effects was much higher than for the commercially available products such as margosan-O, active at a 3.75 ppm azadirachtin level [22]. However, it was comparable with another commercially product toosendanin at 20 ppm [23]. The three compounds were evaluated for feeding deterrent activity against stored product insect pests for the first time.

**Table 1:** Feeding deterrent activity of the pure compounds isolated from *C. tagal* stems and twigs against *T. castaneum *adults.

Treatment	Concentration (ppm)	Consumption of diet * (% control ± SD)	EC_50 _(95% FL)	Slope ± SD	Chi square (χ^2^)
Control		100.00 ± 4.83a	-	-	
Tagalsin A	1000	40.05 ± 3.78e			
	300	53.15 ± 4.23d	375.3	2.43 ± 0.16	27.38
	100	73.43 ± 4.89c	(327.8-428.8)		
	30	88.28 ± 3.674b			
	10	97.56 ± 2.79ab			
Tagalsin B	1000	38.85 ± 4.12e			
	300	47.65 ± 4.33d	277.6	2.64 ± 0.15	19.36
	100	65.32 ± 5.02c	(245.8-309.3)		
	30	84.17 ± 3.43b			
	10	95.23± 3.78a			
Tagalsin H	1000	35.41 ± 4.57e			
	300	49.12 ± 5.03d	285.4	2.56 ± 0.14	32.67
	100	63.26 ± 4.09c	(244.9-327.1)		
	30	73.72 ± 4.14b			
	10	92.86 ± 3.24a			

* Multiple range test using Tukey’s test (P < 0.05). Within each compound, the same letters denote treatments not significantly different from each other.

Dietary tagalsin A, B and H possessed feeding deterrent activity against *T. castaneum *adults (EC_50_ = 375.3, 277.6 and 285.4 ppm, respectively). When compared with the commercial feeding deterrent, toosendanin, the three isolated compounds were 4–5 times less active against *T. castaneum* adults because toosendanin exhibited feeding deterrent activity against *T. castaneum *adults with an EC_50_ value of 66 ppm [23,24]. In the previous report [20], tagalsins Q, R, and U also isolated from *C. tagal* the stems and twigs showed moderate antifeedant activity against the third-instar larvae of *Brontispa longissima* at a concentration of 1 mg/mL.

## 3. Experimental

### 3.1. Plant Material

Fresh stems and twigs (10 kg) of *C. tagal* were collected at the mangrove garden in Hainan Island (20.02° N latitude and 110.20° E longitude), China, in September 2010. The stems and twigs were air-dried (7.8 kg) and ground to a powder using a grinding mill (Retsch Muhle , Germany). The species was identified, and the voucher specimens (BNU-HSL-Dushuahan-2010-09-15-003) were deposited at the Herbarium (BNU) of College of Life Sciences, Beijing Normal University.

### 3.2. Insects

The red flour beetle, *T. castaneum* were obtained from laboratory cultures maintained for the last 10 years in the dark in incubators at 28–30 °C and 70%–80% relative humidity. *T. castaneum* was reared on wheat flour mixed with yeast (10:1, w:w). Adults of *T. castaneum* used in all the experiments were about 2 weeks old.

### 3.3. Extraction and Isolation of Active Ingredients

The powdered stems and twigs of *C. tagal *were extracted with 95% ethanol (100 L) at room temperature over a period of three weeks, and the extract was evaporated under reduced pressure using a vacuum rotary evaporator to afford a syrupy gum (324 g). This syrup was partitioned between methanol-water and petroleum ether (3 × 5,000 mL). The petroleum ether extracts were evaporated off to give a residue (35 g). The aqueous layer was re-partitioned with chloroform (3 × 5,000 mL) to provide a residue (187 g) after evaporation of chloroform. Further partitioning with ethyl acetate (3 × 5,000 mL) gave a residue (96 g) after evaporation of ethyl acetate. Based on the previously described bioassays, only the petroleum ether extracts exhibited strong antifeedant action and were chosen for further fractionation. The petroleum ether residue (35 g) was applied to a silica gel column (160–200 mesh, Qingdao Marine Chemical Plant, Shandong Province, China), eluting with petroleum ether containing increasing accounts of ethyl acetate (from 100:1 to 1:2) to give thirteen combined fractions according to TLC detection. Based on the previously described bioassays, fractions 3 and 5 were chosen for further purification. Fraction 3 (0.8 g) was subjected to a Sephadex LH-20 column (18–110 μm, Pharmacia) and eluted with CHCl_3_-MeOH (1:1) to yield tagalsin H (13.0 mg). Fraction 5 (64 mg) was subjected to silica gel column and eluted with petroleum ether-acetone (8:1) to afford tagalsins A (15.6 mg) and B (17.5 mg). Tagalsin A was recrystallized as needles from acetone. The structures of the compounds were elucidated based on mass spectrometry and nuclear magnetic resonance.

### 3.4. Feeding Deterrent Activity

A flour disk bioassay was used to direct the isolation of active compounds from *C. tagal* extracts according to the method of Xie *et al*. [22] with some modifications [1,24]. Wheat flour (1.0 g) was ultrasonically stirred in distilled water (5 mL) and ethanol (50 µL) containing a fraction or pure compound was added. Pure compounds were first dissolved in ethanol (500 μL) and two drops of Tween-20 (approximately 50 μg) were added to the wheat flour suspension. Aliquots (200 μL) of this stirred suspension were placed on the bottom of a polystyrene Petri dish to form disks. The pipette was fitted with a disposable tip that had an opening enlarged to about 2 mm internal diameter by cutting about 1 cm from the bottom of the tip with a razor blade. The same amounts of ethanol and Tween-20 were applied to produce the control flour disks. The flour disks were left in the fume-hood overnight to air dry. The flour disks were then transferred to an incubator to equilibrate at 28–30 °C and 70%–80% R.H. for 48 h. Each flour disk weighed between 36 and 39 mg. The moisture content of the disk was determined to be 13.5 ± 0.1% using the Kett’s Grain moisture tester (Model PB-1D2, Japan). The disks were placed in glass vials (diameter 2.5 cm, height 5.5 cm) for weighing. Twenty group-weighed, unsexed insects were then added to each vial prior to further weighing. All the insects were starved for 24 h before use. Six replicates were carried out for all treatments and controls. The experimental set-up was left in the incubator for 3 days. Finally, the uneaten parts of the flour disks were weighed. The insect consumption for the different test substances was compared to the control group. Glass vials containing treated flour disks but without insects were prepared to determine any decrease in weights that might have occurred due to evaporation of solvents. Extracts/fractions were tested feeding deterrent activity at a concentration of 1,000 ppm in bioactivity-guided fractionation.

### 3.5. Apparatus

Melting points were measured on a Buchi 535. ^1^H- and ^13^C-NMR spectra were recorded on a Bruker Avance DRX 500 instrument using CDCl_3_ as solvent with TMS as internal standard. EI-MS were determined on an ThermoQuest Trace 2000 mass spectrometer at 70 eV (probe), ESI-MS were determined on a Finnigan LCQ mass spectrometer.

### 3.6. Compound Characterization

*Tagalsin *A. Pale yellow needle crystals. m.p. 68–71 °C [67–69 °C (11)]. EI-MS *m/z* (%): 316 [M]^+^ (15), 286 (17), 259 (20), 179 (27), 163 (53), 136 (100), 107 (93), C_20_H_29_O_3_. ^1^H-NMR δ (ppm): 6.35 (1H, d, *J* = 6.8 Hz, H-1), 5.80 (1H, dd, *J *= 10.5, 17.5 Hz, H-15), 4.93 (1H, d, *J* = 17.5 Hz, H-16), 4.85 (1H, d, *J* = 10.5 Hz, H-16), 3.43 (1H, d, *J* = 6.0 Hz, H-18), 2.95 (1H, d, *J* = 6.0 Hz, H-18), 2.15 (1H, d, *J* = 6.8 Hz, H-10), 1.52 (1H, ddd, *J* = 3.0, 4.0, 12.5 Hz, H-11), 1.48 (2H, m, H-7, 12), 1.45 (1H, m, H-6), 1.41 (1H, dd, *J* = 10.5, 12.5 Hz, H-11), 1.34 (1H, dd, *J* = 11.5, 12.5 Hz, H-14), 1.21 (1H, dd, *J* =3.0, 14.0 Hz, H-12), 1.18 (1H, m, H-6), 1.17 (1H, s, H-19), 1.06 (1H, m, H-7), 1.05 (1H, m, H-17), 1.02 (1H, m, H-14), 0.79 (1H, s, H-20). ^13^C-NMR δ (ppm): 190.8 (C-3), 150.3 (C-15), 146.9 (C-2), 120.8 (C-1), 108.5 (C-16), 60.3 (C-4), 54.5 (C-10), 50.4 (C-18), 40.6 (C-8), 39.8 (C-9), 39.5 (C-14), 36.4 (C-13), 35.9 (C-5), 34.9 (C-11), 33.9 (C-6), 31.8 (C-12), 31.6 (C-19), 27.3 (C-7), 22.8 (C-17), 12.1 (C-20). The ^1^H and ^13^C-NMR data were in agreement with the reported data [11].

*Tagalsin *B. White solid. m.p. 66–69 °C [66–68 °C (11)]. EI-MS *m/z* (%): 316 [M]^+^ (17), 283 (20), 255 (18), 175 (49), 136 (63), 107 (100), 81 (69), 67 (43), 55 (50), C_20_H_29_O_3_. ^1^H-NMR δ (ppm): 6.31 (1H, d, *J* = 6.5 Hz, H-1), 5.87 (1H, dd, *J* = 10.5, 17.5Hz, H-15), 4.92 (1H, d, *J* = 17.5 Hz, H-16), 4.85 (1H, d, *J* = 10.5 Hz, H-16), 3.13 (1H, d, *J* = 6.0 Hz, H-18), 3.09 (1H, d, *J *= 6.0 Hz, H-18), 2.19 (1H, d, *J* = 6.5 Hz, H-10), 1.60 (1H, m, H-6), 1.56 (1H, m, H-11), 1.46 (2H, m, H-8, 12), 1.43 (1H, m, H-11), 1.34 (1H, dd, *J* = 13.0, 13.5 Hz, H-14), 1.25 (1H, m, H-12), 1.23 (1H, m, H-7), 1.21 (1H, s, H-19), 1.18 (2H, m, H-6, 7), 1.05 (1H, s, H-17), 0.72 (1H, s, H-20). ^13^C-NMR δ (ppm): 191.8 (C-3), 150.3 (C-15), 147.3 (C-2), 120.2 (C-1), 108.6 (C-16), 61.3 (C-4), 54.8 (C-10), 55.4 (C-18), 40.1 (C-8), 39.3 (C-14), 39.0 (C-9), 36.9 (C-5), 35.9 (C-13), 34.7 (C-11), 31.9 (C-6), 31.7 (C-12), 29.5 (C-19), 27.0 (C-7), 22.7 (C-17), 13.1 (C-20). The ^1^H and ^13^C-NMR data were in agreement with the reported data [11].

*Tagalsin *H. white powder. m.p. 103–105 °C [101–102 °C (11)]. ESI-MS *m/z*: 305.18 [M]^+^, C_19_H_29_O_3_. ^1^H-NMR δ (ppm): 5.80 (1H, dd, *J* = 10.5, 17.5 Hz, H-15), 4.88 (1H, d, *J* = 17.5 Hz, H-16), 4.85 (1H, d, *J* = 10.5 Hz, H-16), 3.13 (1H, dd, *J* = 2.0, 17.0 Hz, H-1), 2.66 (1H, dd, *J* = 6.0, 17.0 Hz, H-1), 2.30 (1H, m, H-6), 2.20 (1H, m, H-18), 1.87 (1H, m, H-10), 1.52 (2H, m, H-8, 12), 1.50 (1H, m, H-11), 1.45 (1H, m, H-7), 1.39 (1H, m, H-14), 1.30 (1H, m, H-6), 1.21 (1H, m, H-12), 1.19 (1H, m, H-7), 1.15 (1H, s, H-19), 1.05 (1H, s, H-17), 1.01 (1H, m, H-14), 0.61 (1H, s, H-20). ^13^C-NMR δ (ppm): 213.9 (C-4), 180.1 (C-2), 160.3 (C-15), 108.6 (C-16), 54.3 (C-10), 50.8 (C-5), 42.4 (C-8), 39.3 (C-14), 39.0 (C-6), 38.7 (C-9), 35.9 (C-13), 33.7 (C-11), 31.9 (C-12), 31.6 (C-1), 28.5 (C-19), 27.7 (C-18), 27.2 (C-7), 23.0 (C-17), 12.1 (C-20). The ^1^H and ^13^C-NMR data were in agreement with the reported data [11,20].

### 3.7. Data Analyses

Analysis of variance (ANOVA) and Tukey’s test were conducted by using SPSS 10 for Windows 98. Percentage of feeding deterrent index was subjected to an arcsine square-root transformation before ANOVA and Tukey’s tests. The EC_50_ (the concentration needed to inhibit insect feeding by 50% relative to controls) was determined by linear regression [25].

## 4. Conclusions

Based on mass screening of medicinal herbs, the ethanol extract of *C. tagal* stems and twigs was found to possess feeding deterrent activity against the red flour beetles (*T. castaneum*). Three feeding deterrent compounds were isolated and identified from the ethanol extract of *C. tagal *by bioactivity-guided fractionation. The concentration used in this study to observe feeding deterrent effects was comparable with that of the commercial product toosendanin. Dietary tagalsin A, B and H possessed feeding deterrent activity against *T. castaneum* adults, but the three isolated compounds were 4–5 times less active when compared with toosendanin. These findings suggest that the ethanol extract of *C. tagal *stems and twigs and three isolated compound show potential for development as natural feeding deterrents for the control of stored product insects.
